# Cluster oligonucleotide signatures for rapid identification by sequencing

**DOI:** 10.1186/s12859-018-2363-3

**Published:** 2018-10-29

**Authors:** Manuel Zahariev, Wen Chen, Cobus M. Visagie, C. André Lévesque

**Affiliations:** 1 Skwez Technology Corp, Box 3674, Garibaldi Highlands, BC, V0N 1T0 Canada; 2 Ottawa R&D Centre, Agriculture & Agri-Food Canada, 960 Carling Ave., Ottawa, ON, K1A 0C6 Canada; 3 Sidney Laboratory Project - Science, Canadian Food Inspection Agency, Floor 2E, Room 233, 59 Camelot Drive, Ottawa, ON, K1A 0Y9 Canada; 4 The Agricultural Research Counci –PPRI, P/Bag X134, Queenswood, 0121 South Africa

**Keywords:** DNA hybridization, Oligonucleotide signatures, Metabarcoding, Metagenomics, Regulated pathogens

## Abstract

**Background:**

Oligonucleotide signatures (signatures) have been widely used for studying microbial diversity and function in wet-lab settings, but using them for accurate *in silico* identification of organisms from high-throughput sequencing (HTS) data is only a proof of concept. Existing signature design programs for sequence signatures (signatures matching exactly one sequence) or clade signatures (signatures matching every sequence in a phylogenetic clade) are not able to identify all possible polymorphic sites for sequences with high similarity and perform poorly when handling large genome sequencing datasets.

**Results:**

We introduce cluster signatures: subsequences that match perfectly and exclusively any group of sequences in a data set. Cluster signatures provide complete recall for primer/probe design and increased discrimination between sequences beyond that of clade signatures. Using cluster signatures for *in silico* identification of HTS targets achieves good precision/recall and running time performance. This method has been implemented into an open source tool, the Automated Oligonucleotide Design Pipeline (adop), included in supplementary material and available at: https://bitbucket.org/wenchen_aafc/aodp_v2.0_release.

**Conclusions:**

Cluster signatures provide a rapid and universal analysis tool to identify all possible short diagnostic DNA markers and variants from any DNA sequencing dataset. They are particularly useful in discriminating genetic material from closely related organisms and in detecting deleterious mutations in highly or perfectly conserved genomic sites.

## Background

Biodiversity research and survey require accurate identification of organisms from the environment, especially those of public concerns, e.g. quarantine species and select agents monitored by national biosafety and biosecurity programs. Identifying the sequences, e.g. DNA markers or genome regions, of concern in ecosystems is the fundamental strategy [1], especially in the metagenomics era which requires high-throughput processing without compromising accuracy and sensitivity.

A widely used strategy for taxonomic assignment of shotgun metagenomes or metabarcodes is to bin [2, 3] or cluster [4, 5] sequencing reads followed by comparing with reference databases using string search algorithms, which, as reviewed previously [6] either depend on alignment-based phylogenetic distances (homology-search), such as BLAST [7] and HMMER [8–13] or k-mer frequency profile (composition) comparison, such as USEARCH [4, 14–20]. These algorithms are implemented in "off-the-shelf" suites for classification of HTS data, such as ShortBRED [21], PanPhlAn [22], MIDAS [23] and mOTU [24], developed for taxonomic classification or for identifying gene homologs from HTS data.

Aligners using BLAST to map reads are very precise, but with high computational cost, while composition-based programs and aligners using suffix-prefix tries are fast but can be imprecise, compounding errors present in most HTS techniques. For example, the average classification accuracy for all fragments of 16S rRNA genes longer than 100 bp was 70% using the Ribosomal Database Project (RDP) Classifier, a text-based Bayesian classifier [17]. A recent study using the same classifier could classify metabarcodes of the 16rRNA genes to family and genus levels with accuracy 75% or lower [25]. Sigma [6] and Pathoscope [10, 26] are systems developed for subspecies and strain-level inference of metagenomics data, but are not applicable to metabarcoding data, since DNA barcodes are known to lack discriminating power for many taxon lineages [27–30]. For instance, the internal transcribed spacer 1 (ITS1) of *Tilletia indica*, a quarantine pathogen in many countries, and *T. walkeri* which is not regulated by most countries except for South Korea, differ only by two bases.

The Minimum Entropy Decomposition (MED) algorithm implemented in OligoTyping [31, 32] identifies information-rich polymorphic sites and iteratively partitions a set of metabarcodes to homogeneous operational taxonomic units (OTUs), until the Shannon entropy profile of a given node is converged or below a given threshold.

Eren et al. [31] stated that MED was able to discriminate taxa with less than 1% sequence variance and is computationally efficient. While MED is excellent in identifying distinct subgroups of a taxon adapted to specific environmental niches, it works best on abundant OTUs/taxa observed across diverse ecosystems, while many pathogens present as rare taxa in the environment. In addition, alignment is required prior to MED when differences in sequence length do not represent biologically meaningful variation, which can be a main constraint on efficiency when processing HTS reads not of the same length, e.g. quality trimmed Illumina data or 454 pyrosequencing data. While the discriminating positions identified by MED have the potential for strain-typing microoganisms, MED does not directly extract oligonucleotide signatures associated with these positions that may be used as primers or probes for the development of molecular diagnostic assays.

Oligonucleotide signatures (**signatures**) are short sequence strings (*λ*-mers) of fixed length (signature length *λ*), normally 18 to 100 bp, that match exactly and exclusively one or more targeted sequence(s) (targets) in a given genetic data set, usually from the same region of the genomes of targeted taxa. Most existing approaches only design **sequence signatures**, i.e. signatures for single sequences^1^ [33–35] or a single group of sequences per run [36, 37] as reviewed previously [38–40].

A few applications were developed to design signatures for pre-defined groups of genomes [41, 42], gene families [43] or **clade signatures**^2^, i.e. signatures for a single phylogenetic clade^3^ [38]. However, these applications either suffer from memory and runtime issues, or are part of larger, special purpose systems [39].


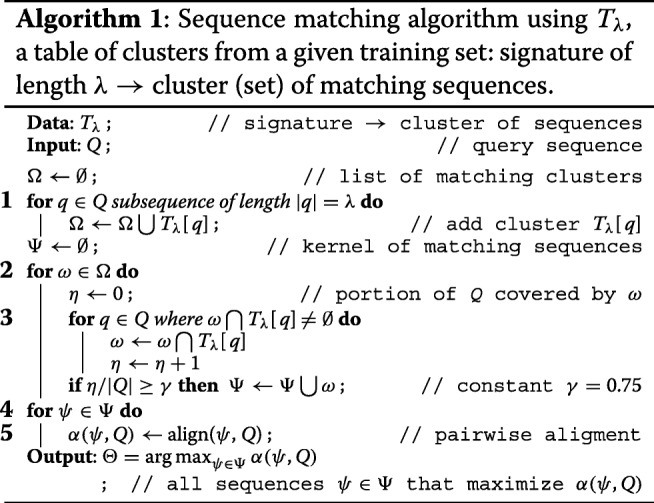


In addition, phylogenetic clades and other a priori groupings can be very restrictive to the identification of viable signatures, which may be caused by conflicting phylogenetic signals among loci shared by different taxonomic domains [44] as found in our own studies [45, 46]. This restrictiveness is further compounded by additional experimental constraints, such as primer/probe melting temperature [47] or Kane’s conditions [48–50].

Signatures have wide applications in the biological field, such as being used as primers and probes in PCR and DNA-hybridization [40, 46, 51–53] or lab-on-a-chip detection methods, as well as in targeted enrichment methods for focused high-throughput sequencing (HTS) [54, 55]. Kallisto [56] uses signatures (of length *k*: *k*-mers) from RNA-Seq reads to create *k*-compatibility classes, whose intersection represents the set of possible sequences matching a given read. Similarly, Salmon [57] builds equivalence classes over fragments of reads (in effect signatures), from which it infers statistically the relative abundance of transcripts. Kraken [58] infers the taxonomic classification for HTS reads by building a database of phylogenetic lowest common ancestors using clade signatures. We show further that a significant number of signatures cross clade boundaries. We also show that while signatures work very well on perfectly preserved reads, they are brittle to errors introduced by the HTS process.

Signatures have also been used to detect pathogenic microbes from metagenomics sequencing data. This theoretical approach, termed Electronic probe Diagnostic Nucleic acid Analysis (EDNA) [59, 60], shows promising research and diagnosis direction (75% precision on a mock database) using shotgun metagenomics data, but relies on a priori groupings of the training data set (reference genomes), and a priori differentiation against false positives identified using near neighbor comparisons in a reference database. EDNA also depends on an external program for signature design, the Tool for Oligonucleotide Fingerprint Identification (TOFI) [42], which introduces runtime efficiency constraints. A system that can streamline this process would be ideal as a regulatory tool in pest detection and management.

We present here a research tool for unrestricted design of signatures that can be used for the detection of any kind of groups (mutants, species/subspecies, or any a priori groupings) in a wide range of molecular biology assays or DNA sequence data for *in silico* probing.

## Methods

The usefulness of signatures is based on the low probability of accidental match between a signature and unrelated genetic material. The probability *p* of an accidental match (collision) between two 4-base nucleotide strings of length *λ* (4^*λ*^ possibilities) in a data set of size *N* can be modeled by the birthday formula [61]:

1 $$ p = \frac{4^{\lambda} !}{4^{\lambda N} \left(4^{\lambda}-N\right)!} \gtrapprox 1 - e^{-\frac{N^{2} }{ 2 \cdot 4^{\lambda}}} \textrm{ for} N \ll 4^{\lambda}  $$

Accidental matches between portions of nucleotide strings occur in random genetic material when there is no taxonomic or functional relationship between the query and testing sequences. For example, it is likely to encounter the subsequence of length *λ*=4 “ACGT” multiple times in different sequences in any given large reference database. The birthday formula quantifies the probability of such accidental matches.

Assuming an uniform distribution of nucleotides and signature length *λ*=36, *p*<10^−4^ applies for data sets with *N*<2.94×10^10^ nucleotides^4^ (approximately 274 GiB of unaligned FASTA files^5^). In practice, data sets of taxonomically related DNA have a higher degree of similarity between sequences, which increases the probability that any two identical subsequences have a taxonomic or functional relationship between them and do not represent accidental matches. Unless explicitly specified, signatures of length *λ*=36 are used for analyses in this study.

### Clusters

We introduce an extension of clade signatures: for a given set of sequences, a **cluster** is a group of sequences for which at least one signature (**cluster signature**) can be found, that matches all sequences in the group but does not match any sequences not in the group.

Notably, clusters are not required to represent the same groups as those in phylogenetic clades; they are *any* groups of sequences for which signatures can be found, as opposed to clade signatures for predefined phylogenetic groups. Any subsequence of length *λ* of any sequence is a signature for exactly one cluster, i.e. a cluster signature.

It is not obvious how to predict the number of clusters expected for a given data set of taxonomically related sequences. For example, *S* identical sequences of length *L*_*i*_>*λ* where 1≤*i*≤*S*, will generate exactly 1 cluster. Since any subsequence of length *λ* from any sequence can be found in every other sequence, the single cluster will contain all sequences. By contrast, a data set with size *N*≪4^*λ*^ containing *S* sequences randomly generated using a uniform distribution of nucleotides will have *S* clusters of signatures of length *λ*. Each such cluster will contain one sequence, since it is very likely that every subsequence of length *λ* in any sequence is a sequence signature: it will not be found anywhere else in the data set *p*<10^−4^ (Eq. 1).

### The automated oligonucleotide design pipeline

We have built an open source tool, aodp, the Automated Oligonucleotide Design Pipeline (aodp v.2.5 is included in the supplementary material), which generates efficiently signatures for sequences, clades and clusters by enumerating all *λ*-mers (signatures) for each sequence in a given data set. The list of originator sequences is collected for each enumerated signature. All distinct sets of originator sequences for all signatures form the list of clusters for the data set. Facilities for enumerating and cross-referencing signatures and clusters are provided.

Furthermore, aodp can be used to find the closest matching sequences from a training set to a query sequence, assumed to be an imperfectly recovered portion of an unknown sequence (such as an HTS read) by computing the union of all sequences in all clusters matching any portion of the HTS read and then heuristically eliminating all but sequences that explain the largest portion of the HTS read. All remaining sequences are then compared to the HTS read and only the ones with the highest overlap score are kept.

More formally, the matching algorithm 1 works as follows: first, compute a set of matching clusters *Ω* for each query sequence *Q* (loop 1): we observe later (Table 3) that the set of all training sequences contained in all matching clusters *Ω* has average size $\overline {\Omega } < S$ smaller than the size of the training set; second, minimize a subset (kernel) *Ψ* of *Ω* (loop 2): we observe that the average size of the kernel $\overline {\Psi } \ll S$ is much smaller than the size of the training set; and, finally, compute the sequence similarity of each training sequence in the kernel *Ψ* against the query sequence (loop 4) using a global alignment algorithm [62].

The result (the set of mapped HTS reads) is the subset of sequences of *Ψ* which maximize the alignment score to the query sequence.

The main objective of the algorithm is to minimize the number of computationally expensive global alignments (step 5). The complexity has no direct dependency on the size of the training set: loops 1 and 3 have complexity *O*(|*Q*|) linear in the size of the query sequence and loop 2 has complexity *O*(|*Ω*|) linear in the number of clusters matching the query sequence, which can be further reduced at the implementation level through the elimination of repeated set operations in loop 3.

A general limitation of algorithms for matching HTS reads (including our method) is that metabarcoding regions used in HTS do not always have sufficient discriminating power to differentiate very closely related species [28] represented by clades in a training dataset containing almost identical sequences, which, however, belong to multiple valid species. In this case, all matched reference sequences are given to a query sequence, and it should be the users’ decision if alternative DNA markers or wet lab molecular diagnostic assays should be used to confirm or validate the existence of targeted taxa of interest.

### Data sets

Data sets for four important mycotoxin genera (*Alternaria*, *Aspergillus*, *Claviceps* and *Penicillium*) were built using the following methodology: internal transcribed spacer rDNA region (ITS) data sets were compiled from GenBank [63] using ex-type sequences as backbone when available and building up the database from additional trustworthy taxonomic reviews [64, 65, 65–68]. The data sets were aligned in MAFFT v. 7.305b [69], using the G-INS-i algorithm and trimmed manually in Geneious v. 8.1.8. Neighbour-Joining trees were calculated in PAUP* v. 4.0b10 [70].

Reference ITS sequences for fungi (*Anisogramma*, *Ceratorhiza*, *Ceratocystis*, *Colletotrichum*, *Coniella*, *Diaporthe*, *Fusarium*, *Elsinoe*, *Talaromyces*, *Tillletia*), oomycetes (*Peronospora*), as well as the 16S rRNA genes of a bacterium (*Pectobacterium*) were downloaded from GenBank. The ITS dataset *Phytophthora* was obtained from [46]. The sequences for each dataset were aligned using the G-INS-i algorithm in MAFFT [69], and trimmed manually in BioEdit v.7.2.5 [71]. The approximate maximum likelihood trees were reconstructed using FastTree v.2.1.8 [72].

Each data set contains DNA sequences and a phylogenetic tree with the sequences as leaf nodes. The data sets were combined into a sequence database *17DataSets*, provided as supplementary material. Sequences with more than five ambiguous bases were removed from each data set. The characteristics of each data set are summarized in Table 1.

**Table 1:** Data sets included in database 17*DataSets*

Data set	*N*	*S*	*i*	*n*	$\bar {L} \pm \sigma (L)$		*n* ^∗^	*c*	*c*/*n*	*n*^∗^/*n*	*s*_0_		*s*_*s*_		*s*_*n*_		*s*_*c*_		*δ*_*c*_
*Anisogramma*	15248	28	26	54	545	94	33	139	2.6	61%	1	4%	24	86%	27	96%	28	100%	4%
*Pectobacterium*	72624	37	42	79	1671	290	43	258	3.3	54%	-	-	25	68%	28	76%	35	95%	19%
*Ceratorhiza*	24645	37	35	72	647	60	36	137	1.9	50%	7	19%	24	65%	25	68%	34	92%	24%
*Coniella*	23078	48	46	94	481	64	45	143	1.5	48%	7	15%	32	67%	37	77%	48	100%	23%
*Talaromyces*	54964	88	86	174	625	220	126	626	3.6	72%	-	-	87	99%	88	100%	88	100%	-
*Elsinoe*	79740	132	63	195	586	146	54	199	1.0	28%	1	1%	37	28%	40	30%	43	33%	2%
*Claviceps*	77453	140	139	279	553	45	92	376	1.3	33%	16	11%	58	41%	63	45%	82	59%	14%
*Ceratocystis*	112291	193	179	372	582	205	115	631	1.7	31%	52	27%	74	38%	82	42%	149	77%	35%
*Phytophthora*	201815	253	238	491	798	24	319	1103	2.2	65%	-	-	149	59%	166	66%	184	73%	7%
*Diaporthe*	213202	399	338	737	530	99	196	1008	1.4	27%	149	37%	140	35%	150	38%	266	67%	29%
*Peronospora*	428994	513	400	913	824	377	349	1984	2.2	38%	64	12%	200	39%	222	43%	310	60%	17%
*Alternaria*	280418	551	550	1101	509	11	187	734	0.7	17%	-	-	78	14%	86	16%	101	18%	3%
*Aspergillus*	547127	1032	1032	2064	530	39	591	2331	1.1	29%	19	2%	285	28%	313	30%	414	40%	10%
*Colletotrichum*	691867	1198	918	2116	576	297	477	2010	0.9	23%	562	47%	379	32%	397	33%	667	56%	23%
*Tilletia*	743335	1200	915	2115	618	259	574	2666	1.3	27%	394	33%	376	31%	403	34%	649	54%	20%
*Penicillium*	743954	1438	1437	2875	517	12	597	2675	0.9	21%	57	4%	310	22%	325	23%	413	29%	6%
*Fusarium*	1604775	2946	2261	5207	533	133	1165	4417	0.8	22%	1492	51%	969	33%	1001	34%	1778	60%	26%

*N*: size of data set (nucleotides), *S*: number of sequences (other than sequences with more than 5 ambiguous bases), *i*: number of internal clades in the phylogenetic tree, *n*: total number of phylogenetic clades *n*=*S*+*i*, $\bar {L}$: average length of sequences in the data set (rounded to closest integer), *σ*(*L*): corrected sample standard deviation for the sequence length (rounded to closest integer). *n*^∗^: number of signable clades, *c*: number of clusters (*λ*=36) identified by aodp, *c*/*n*: ratio between clusters and phylogenetic clades, *n*^∗^/*n*: ratio between signable clades and phylogenetic clades, *s*_0_: number of sequences that are not included in any signable clades, *s*_*s*_: signable sequences (also unique signable sequence patterns), *s*_*n*_: unique signable clade patterns, *s*_*c*_: unique cluster patterns, *δ*_*c*_=*s*_*c*_−*s*_*n*_: discrimination increase attributable to clusters (difference between unique cluster patterns and unique signable clade patterns)

The distribution of cluster size and number of cluster signatures was also studied on a much larger dataset (*Unite*; included in the supplementary material) of 271,017 sequences fully identified down to the species level and which include an authoritative Latin binomial name for each species. The data set was extracted from the UNITE+INSD database released by the User-friendly Nordic ITS Ectomycorrhiza Database (UNITE, version 7.1^6^), [73]. A phylogenetic tree was automatically built from the Unite taxonomy using tax2nwk, a companion utility of aodp.

The sequence matching functionality was evaluated using a training set of 1,338 mycotoxin sequences (*4Mycotoxins*; included in the supplementary material) by combining the data sets *Alternaria*, *Aspergillus*, *Claviceps* and *Penicillium*. Sequences from each data set not classified to the principal genus of the data set and/or with more than five ambiguous bases were eliminated.

The precision and recall of the matching algorithm were evaluated using a testing set *4MicotoxinsBootstrap* bootstrapped from *4Mycotoxins*: subsequences of exactly |*Q*|=100 bp starting at a random position were extracted from each sequence. In each subsequence, each nucleotide was modified to another nucleotide or a gap. Individual modifications were made at one of six error rates: *ε*∈{0.00, 0.01, 0.02, 0.03, 0.04, 0.05 }. For each sequence and each error rate, 10 subsequences were generated. A total of 80,280=1,338×6×10 query sequences were generated. All random choices were drawn from uniform distributions driven by a Mersenne twister [74], seeded with a high resolution timestamp.

The efficiency of the matching algorithm was evaluated on a testing set *97AerobiotaSamples* containing 4,713,791 sequences (sequence length |*Q*|≈436bp±55; only sequences at least 325 bp long are selected) from a data set deposited in the Sequence Read Archive (SRA) under project accession number PRJNA358221. The error rate assigned to the data set was *ε*=0.01 [75].

### Comparisons with other algorithms

We have compared the computational efficiency of our matching algorithm with BLAST+ v.2.6.0 [7] and USEARCH v10.0.240_i86linux32 [4] testing on the *97AerobiotaSamples* data set and using the *4Mycotoxins* reference data set. All test runs were conducted on a system with Intel Core i7-3632QM CPU 2.20GHz ×8 running Ubuntu 16.04.

The following parameters were used for BLAST: “-word_size 11 -outfmt 6 -num_threads 8 -evalue 10 -max_target_seqs 100”.

The following parameters were used for USEARCH: “-usearch_global -strand plus -id 0.98 -maxaccepts 256 -maxrejects 1024 -wordlength 8 -blast6out”.

In all instances, output was ignored (redirected to /dev/null) in order to eliminate I/O contention.

Separately, we compared precision and recall (Eqs. 2 and 3) of our matching algorithm with USEARCH, on the *4MycotoxinsBootstrap* dataset using the *4Mycotoxins* reference set.

For USEARCH we used the following parameters: “-usearch_global -strand plus -wordlength 8 -blast6out”. Additionally, the “-id” parameter was set to 1−2*ε* to correspond to the error rate of the data set, “maxaccepts” *χ* was varied for different runs *χ*∈{4,16,64,256,1024} and “maxrejects” was set to 32×*χ*.

For both USEARCH and aodp, the match between a query sequence and a training sequence was considered correct if it is returned by the tool, and it has the highest percentage overlap compared to all other matching training sequences.

## Results

**Fig. 1:**
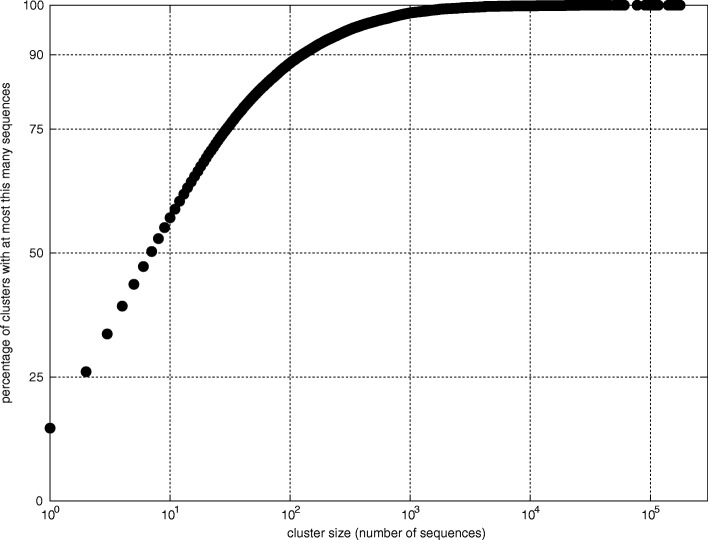
Distribution of number of sequences per cluster (cumulative percentage), data set *Unite*, *λ*=36

**Fig. 2:**
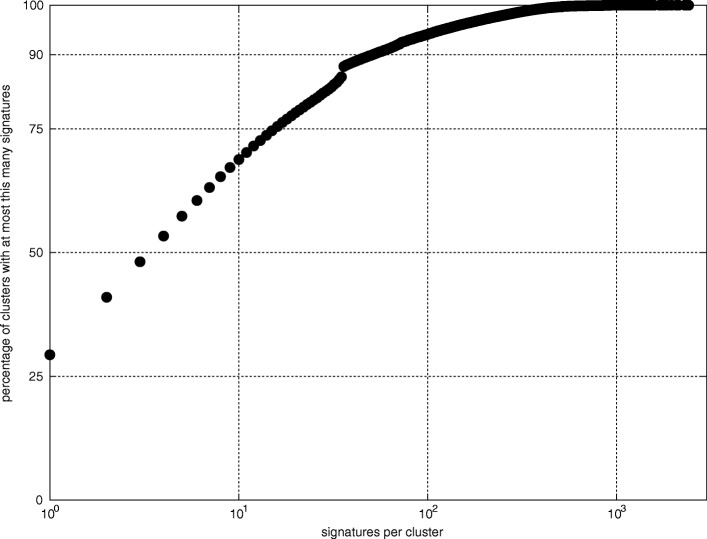
Distribution of number of signatures per cluster (cumulative percentage), data set *Unite*, *λ*=36

Large scale dependencies for the number of clusters were measured on the data set *Unite*. Most clusters have a relatively small number of sequences (Fig. 1): 85% have less than 100 sequences, 50% have less than 10 sequences and approximately 15% have one sequence (signable sequences). Clusters have a relatively small number of signatures (Fig. 2): 65% have less than 10 signatures and almost 30% have exactly one signature.

**Fig. 3:**
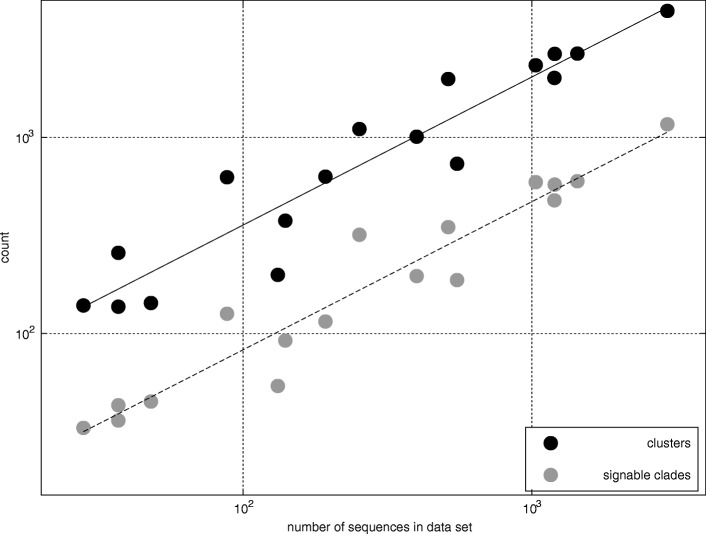
Dependency of the number of clusters (groups of sequences for which at least one signature can be found) and number of signable clades (phylogenetic clades to which oligonucleotide signatures can be assigned) on number of sequences within each dataset (database 17*DataSets*, *λ*=36)

Other dependencies for the number of clusters are measured on the *17DataSets* database. The number of clusters *c* is found to be comparable with the number of phylogenetic clades *n*=*S*+*i* in each of the data sets (0.7 ≤*c*/*n*≤ 3.6). Power law dependencies on the size of the data set *N* for the number of clusters *c* and number of signable clades *n*^∗^ are indicated by a log-log plot (Fig. 3). A power law dependency of the number of clusters *c* on the number of signable clades *n*^∗^ is indicated by regression lines.

**Fig. 4:**
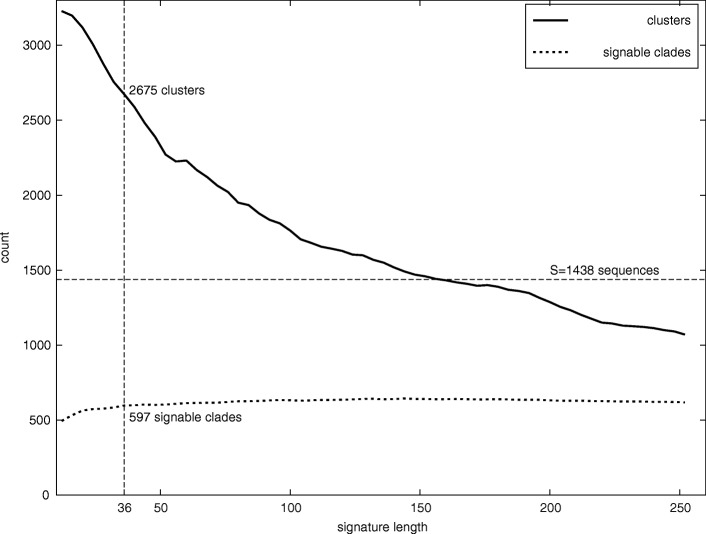
Dependency of the number of clusters (groups of sequences for which at least one signature can be found) and number signable clades (phylogenetic clades to which oligonucleotide signatures can be assigned) on signature length, data set *Penicillium*

The dependency of the number of clusters and signable clades on signature size 12≤*λ*≤252 (increments of 4 nucleotides) is measured for the data set *Penicillium* (Fig. 4). The number of clusters *c* decreases rapidly with the signature length *λ*, because of the further reduction of the number of signatures in each cluster. The number of signable clades is relatively stable (slow initial increase).

### Clusters for probe design

**Fig. 5:**
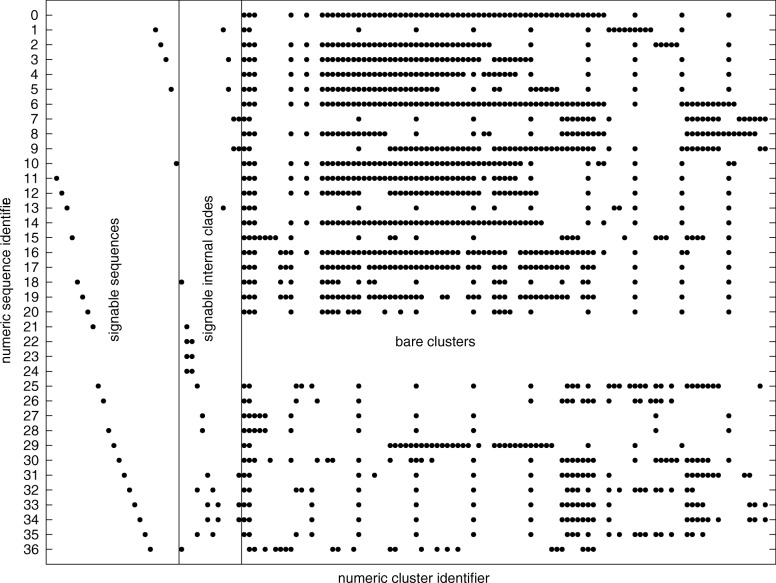
Sequence by cluster incidence matrix for the *Ceratorhiza* data set (*λ*=36). Each row contains cluster matches associated with the sequence with numeric identifier on the y-axis (a *fingerprint* of the sequence). Each column represents sequences contained in a given cluster. Signable sequences and signable internal clades are at the left. The remaining (*bare*) clusters are at the right

Characteristics of clusters, signable clades and signable sequences were calculated in aggregate for all data sets and reported in Table 1. An incidence matrix for sequences (vertical axis) against clusters (horizontal axis) for the *Ceratorhiza* data set is shown in Fig. 5. Signed sequences and internal signable are grouped in regions to the left of the figure.

The number of signable clades is smaller than the number of clades *n*^∗^<*n*, in some cases substantially, for example *n*^∗^/*n*=17*%* for *Alternaria*.

The number of sequences *s*_0_ that are not contained in any signable clades can be substantial. This likely indicates a data set with high degree of similarity between sequences in different phylogenetic clades.

For example, 50% of sequences in the *Fusarium* data set are not contained in any signable clade. For the *Ceratorhiza* data set, seven sequences (0, 4, 6, 8, 14, 16 and 17) have no signable clades (18% of the total).

Since every subsequence of length *λ* of every sequence is a cluster signature, every sequence is a member of at least one cluster. In other words, clusters provide signatures for every sequence of a data set (complete recall).

Each sequence has an associated cluster pattern (fingerprint) in the sequence-to-cluster incidence matrix (Fig. 5). This pattern may be unique for the sequence or can be shared with other sequences. For example, sequence 36 has a unique cluster pattern, but sequences 22, 23 and 24 have identical patterns. We call the number *s*_*c*_ of unique row patterns in the incidence matrix, *unique cluster patterns*.

If only sequence signatures are taken into account, all signable sequences can be uniquely and trivially identified by a sequence signature (a cluster of size 1). In other words, the number *s*_*s*_ of sequences that can be uniquely identified using sequence signatures is the number of signable sequences.

If taking into account signable clades and signable sequences, additional sequences may be uniquely identified from their signable clade pattern. For example, sequence 27 can be uniquely identified using its clade signature pattern, although it could not be identified using its sequence signature (it does not have one). We call the number *s*_*n*_ of such sequences, unique signable clade patterns.

For each data set, the quantities *s*_*s*_, *s*_*n*_ and *s*_*c*_ were computed and the percent change *δ*_*c*_=*s*_*c*_−*s*_*n*_, which we call *discrimination* attributable to clusters. For all data sets except *Talaromyces*, *δ*_*c*_>0. Substantial gains can be seen, for example, for *Ceratocystis* (35%), *Diaporthe* (29%) and *Fusarium* (26%).

For the two data sets with the highest ratio less than 100% of unique signable clade patterns *s*_*n*_: *Anisogramma* (*s*_*n*_=96%) and *Coniella* (77%), the ratio of unique cluster patterns *s*_*c*_ increases to 100%.

Compared to using only phylogenetic clade signatures, where some sequences do not appear in any signable clade (*s*_0_>0 in most cases) recall (Eq. 3) is always 100% when using clusters (since every sequence is a member of at least one cluster). Selectivity is also increased since more sequences can be differentiated through unique cluster patterns vs. unique signable clade patterns (*δ*_*c*_>0 in most cases).

Consequently, the design of wet lab probes based on cluster signatures can improve recall and selectivity compared to designing only sequence or clade signature probes.

### Clusters for high-throughput sequencing

Precision (Eq. 2), recall (Eq. 3) and the *F*-measure (Eq. 4) for the matching algorithm 1 were evaluated using the *4Mycotoxins* training set and the bootstrapped *4MicotoxinsBootstrap* testing set^7^ for signature lengths *λ*∈{8, 16, 24, 32, 40 }. Identification was considered correct if for a query sequence its originator sequence from the training set is reported among the sequences with maximum similarity *α* over a threshold (Eq. 5) dependent on the error rate *ε*.

2 $$\begin{array}{*{20}l} \text{precision} & = \frac{\textrm{correctly identified}}{\textrm{total identified}}  \end{array} $$

3 $$\begin{array}{*{20}l} \text{recall} & = \frac{\textrm{correctly identified}}{\textrm{total number of terms}}  \end{array} $$

4 $$\begin{array}{*{20}l} F & = \frac{2 \times \text{precision} \times \text{recall}}{\text{precision} + \text{recall}}  \end{array} $$

5 $$\begin{array}{*{20}l} \alpha \geq 1 - 2\epsilon  \end{array} $$

Precision and recall was found to decrease with the increase of the error rate *ε* and signature length *λ*. Precision was consistently above 0.9 for *λ*≥16. Recall degraded below 0.5 for higher error rates.

Imperfect recall is due to two factors: “crowding” of defects in a query sequence to the point where there are no preserved subsequences of length *λ*, and to query sequences that have more errors, failing the similarity threshold (Eq. 5).

Imperfect precision is due to errors in the query sequence leading to accidental matches and higher similarity scores (*α*) for sequences in the training set other than the originator sequence. This is likely to happen in training sets with high degree of similarity between sequences, for example, when an error may coincide with a single nucleotide polymorphism site.

Precision and recall for the bootstrapped test sets can be applied to only matching sequences in real test sets, where a substantial portion of the data may be unrelated to the training set.

Precision and recall are driven by the size and nature of the training set and the statistical properties of the error-introducing mechanism.

Precision and recall were compared on the same data sets with USEARCH by varying the *maxaccepts* parameter *χ*∈{4,16,64,256,1024}.

We notice that USEARCH outperforms aodp for the highest value of *χ*=1024 on the combined *F* measure (Eq. 4), however aodp outperforms USEARCH for smaller values of *χ* and at lower error rates *ε*≤0.03. Moreover, the values of *χ* and the related USEARCH parameter “maxrejects” must be chosen a priori. The optimal value of this parameter likely depends on the degree of similarity of sequences within the training set. For example, for *4Mycotoxins*, the optimal value is in range of the total number of sequences (1,338).

For aodp, the optimal value of *λ* can be chosen based on the error rate of the testing set. The set of matching sequences self-calibrates to the size of the matching clusters.

The computational efficiency of the matching algorithm was measured on a realistic test set *97AerobiotaSamples*, for different values of the signature length *λ*∈{16,24,32,40}.

The number *μ*98 of matching query sequences (query sequences with similarity *α*≥1−2*ε*=0.98 to at least one training sequence) is relatively stable for different values of *λ*. The matching kernel *Ψ*≈*Θ* is a close approximation of the result set.

The set of all training sequences contained in all matching clusters *Ω* has average size $\overline {\Omega } < S = \textrm {1,338}$ smaller than the size of the training set. The average size of the kernel $\overline {\Psi } \ll S$ (average number of alignments) is much smaller than the size of the training set, which shows that using clusters and reducing the matching sequence kernel are effective in reducing the number of alignments, and consequently running time compared to a brute force approach that would align every query sequence to every sequence in the training set.

Running time is dependent on the number of alignments ($\overline {\Psi }$), and to the set of training sequences in matching clusters ($\overline {\Omega }$): loop at line 2 in algorithm 1.

Running time degrades to impractical values for lower values of *λ* (estimated at over 200h for *λ*=8). This is due to very large values of $\overline {\Omega }$ (overfitting) from large number of clusters in the training set at low signature length (Fig. 4) and high likelihood of accidental matches between short signatures and query sequences (Eq. 1).

Running time is also measured for BLAST and USEARCH on the same data sets. aodp outperforms BLAST by one order of magnitude. Running time is also faster, if comparable to USEARCH.

## Discussion

The number of clusters is larger than the number of signable clades (Fig. 4), but comparable to the total number of clades. Within experimental constraints, it is feasible to design signatures for each cluster in a data set. Cluster signatures offer increased discrimination compared to sequences or clades signatures.

The number and composition of clusters is an objective property of a given data set. Conversely, phylogenies can be subjective when prepared by human taxonomists or inaccurate when automatically built using specific heuristics, in some cases with subjective parameters.

Most clusters have a small number of signatures (are brittle to additional experimental constraints) and a small number of sequences (have focused discrimination). To achieve optimal discrimination for clusters, signature length should be chosen as small as practical above the lower limit imposed by the birthday formula (Eq. 1).

Clusters provide signatures for every sequence in a data set (complete recall).

This makes it practical to design probes that identify DNA sequences from data sets with very closely related material, where some of the sequences may not be represented in any of the signable clades. Unique cluster patterns associated with sequences (Fig. 5) can help uniquely identify sequences from a data set, beyond the ability of unique signature clade patterns, in some cases for 100% of the sequences.

Cluster signatures can be used as clues for identifying partial, imperfectly copied query sequences (such as produced by HTS) against a training set of reference sequences. Combined with a global alignment algorithm for comparing candidate sequences from the matching sequence kernel of a set of matching clusters, a matching algorithm (algorithm 1) achieves good matching precision and recall for test sets of different quality (Table 2).

**Table 2:** Precision and recall of our matching algorithm (aodp) and USEARCH using the *4Mycotoxins* training set and the *4MicotoxinsBootstrap* testing set

aodp										
*λ*	8	16	24	32	40	8	16	24	32	40
*ε*	Precision					Recall				
0.05	0.74	0.90	0.91	0.92	0.93	0.71	0.49	0.25	0.15	0.08
0.04	0.78	0.92	0.92	0.92	0.93	0.76	0.64	0.38	0.23	0.14
0.03	0.83	**0.95**	0.95	0.95	0.96	0.80	**0.78**	0.55	0.38	0.24
0.02	0.89	**0.97**	0.97	0.97	0.97	0.84	**0.88**	0.74	0.57	0.43
0.01	0.95	**0.99**	0.99	0.99	0.99	0.87	**0.91**	0.88	0.78	0.68
0.00	1.00	**1.00**	1.00	1.00	1.00	1.00	**1.00**	1.00	1.00	1.00
USEARCH										
*χ*	4	16	64	256	1024	4	16	64	256	1024
*ε*	Precision					Recall				
0.05	0.21	0.40	0.62	**0.88**	**0.98**	0.19	0.38	0.60	**0.84**	**0.94**
0.04	0.21	0.41	0.63	**0.89**	**0.98**	0.20	0.38	0.59	**0.83**	**0.92**
0.03	0.21	0.41	0.64	0.89	**0.99**	0.20	0.39	0.60	0.84	**0.93**
0.02	0.22	0.43	0.66	0.91	**0.99**	0.20	0.40	0.61	0.84	**0.92**
0.01	0.24	0.45	0.67	0.92	**1.00**	0.21	0.41	0.61	0.83	**0.90**
0.00	0.28	0.50	0.72	0.95	**1.00**	0.26	0.50	0.72	0.95	**1.00**

Rows have a given error rate *ε* For aodp, columns have a given signature length *λ*. For USEARCH, columns have a given value *χ* for the “maxaccepts” parameter. Cells where USEARCH outperforms aodp on the *F* measure are in **bold**. Cells where aodp outperforms USEARCH on the *F* measure for *χ*≤256 are also in **bold**

Using a set of matching clusters *Ω* to the query sequence significantly reduces the number of pairwise comparisons ($\overline {\Omega }$ Table 3) compared to the brute force approach. Reducing to a kernel of matching sequences $\overline {\Psi }$ further decreases the number of alignments and provides good running time performance, with dependence on the number and size of clusters in the training database, but not on the actual size of the training database.

**Table 3:** Performance of the matching algorithm using the *4Mycotoxins* training set (1,338 sequences) and the *97AerobiotaSamples* testing set by signature length *λ*

aodp					
*λ*	*μ*98	$\overline {\Theta } / \overline {\Psi }$	$\overline {\Psi }$	$\overline {\Omega }$	*t*
16	1352	0.93	0.317	17.41	17039
24	1353	0.94	0.311	13.27	9720
32	1342	0.95	0.299	11.83	6362
40	1325	0.94	0.298	11.06	3031
USEARCH					32560
BLAST					74335

*μ*98: number of matching query sequences with similarity *α*≥1−2*ε*=0.98, *t*: running time in seconds (system description in “Comparisons with other algorithms” section). Average values (algorithm 1) are reported for: size of the matching kernel $\overline {\Psi }$, number of sequences in all matching clusters $\overline {\Omega }$. Ratio $\overline {\Theta } / \overline {\Psi }$: average size of the result set to the average size of the matching kernel. Running times are also reported for USEARCH and BLAST

Increasing the signature length *λ* generally increases the precision and decreases the running time of the matching algorithm, but decreases the recall, even to unsatisfactory values (Table 2) for testing sets with high error rates *ε*. However, lower recall values (e.g. at or below 50%) may be acceptable when the assertion of existence of the target and not the accuracy in abundance was the objective of the investigation. Also, sequencing read accuracy at or above 98% (*ε*≤0.02) is provided by the majority of HTS techniques, although sometimes through building consensus [76].

Choosing very small values for the sequence length (*λ*<16) leads to overfitting.

Additional thermodynamic constraints such as the elimination of homopolymer regions and filtering on melting temperature [47, 77] also apply to the design of signatures for assay development. Because of the variability of study objectives and experimental conditions, thermodynamic constraints have not been taken into account in analyses in this study, although support is built into aodp.

Results reported in the current study were drawn primarily from a wide variety of fungal groups, with a focus on plant pathogen and mycotoxin producers. We are confident that these can be generalized to include sequences from other organisms.

Since clusters do not rely on phylogenetic assumptions, but may only coincide with phylogenetic clades, there is no direct dependency of cluster parameters on a specific phylogeny. The phylogeny independent clusters can be particularly useful when it it important to follow some specific DNA sequences such as resistance point mutations or horizontally acquired genes.

A comprehensive study on a variety of data sets with different length distributions and systematically varied completeness and diversity may provide further insights (future research). Power law goodness-of-fit tests such as the Kolmogorov-Smirnoff statistic [78] for the dependencies of the number clusters and signable clades on number of sequences using a larger number of data sets may help quantify results.

Precision and recall were evaluated on a data set (*4MicotoxinsBootstrap*) of 100bp sequences with randomly-introduced errors at given error rates from a source data set (*4Mycotoxins*). While this does not account for variable read length generated by different HTS methods or for non-random defects, such as homopolymer errors or issues related to palindromic sequences, precision and recall targeted to specific methods can be modeled into the defect-introducing mechanism.

On precision and recall (*F* measure), our matching algorithm outperforms USEARCH for lower values of “maxaccepts” (*χ*≤256) and lower error rates (*ε*≤0.03). This likely happens in situations of closely related portions of training sequences, of which a large number (possibly overlapping a signature cluster) are equally similar to the training sequence. By imposing a limit on the size of the result set, the source sequence may or may not be included in the first *χ* USEARCH matches. Similar behaviour can be expected when varying the “max_target_seqs” in BLAST.

USEARCH outperforms our algorithm for higher values of *χ* (in range of the number of training sequences), but this parameter is dependent on the degree of similarity of the training set and must be chosen a priori. Always choosing large values may be impractical, since the size of the result sets increase dramatically with higher values of *χ*.

Precision and recall were only evaluated on the bootstrapped data set, where introduced errors could be traced back to the originator sequence (ground truth) for comparison with the reported matches. Such a source of ground truth could not be easily derived for the larger *97AerobiotaSamples* data set since the query sequences are, by definition, unknown and the number of matching terms may be too small to draw statistically sound conclusions: est. 1,300 vs. 80,280 terms for *4MycotoxinsBootstrap*. Future research may look at comparisons on a real data set with a substantial number of matches with matches reported by BLAST as a source of ground truth. An additional complication for such a study may be the need to choose a high “max_target_seq” parameter to cover all possible matching training sequences, resulting in very high running times.

Our matching algorithm outperforms BLAST on running time by one order of magnitude on a realistic data set, with a "max_target_seqs" *χ*=100. BLAST performance degrades much further for higher values of *χ*.

aodp also outperforms USEARCH on running time. This may be due to the nature of the testing set: approximately 1,300 matches in about 4.5 million reads, which may be reasonable for targeted studies of environmental samples, but may not hold for other types of investigations.

On a highly parallel system (80 hardware threads; results unreported), the difference increases to two orders of magnitude for BLAST and increses further for USEARCH because of good multithreading scalability of aodp.

The number and size of clusters are fixed parameters of a given database and represent the main drivers for the running time of the matching algorithm. In situations where short running time is essential without access to large computational resources, running time may be shortened by increasing the signature length (*λ*) at the expense of recall, e.g. for a preliminary “quick” run, or the size of the source database can be reduced (which will lead to a reduction of the number and size of clusters).

Further improvement of the running time for aodp may be achieved by a more efficient implementation of the global alignment algorithm (step 5 in algorithm 1), such as using nucleotide k-mers, or alignment clues from the positioning of the matching signatures.

Conversely, cluster signatures could be used in a preprocessing step to quickly eliminate or identify candidate matching sequences, to be further validated using a matching algorithm with different objectives.

The study of the signability of other groupings such as gene function may be useful.

Another promising avenue of future research may be the study of cluster signatures for genetic variants in guiding the detection of mutations relevant to evolution, genetic diseases or rapid comparisons of genomes between tumors and healthy cells.

## Conclusions

In this study, we evaluated the statistical properties of cluster signatures (oligonucleotide signatures for groups of sequences in data sets of DNA sequences) and their use for mass identification by sequencing.

Our method is universal as it can find oligonucleotide signatures for unique strains, species, higher level phylogenetic clades or mutations linked to genetic diseases or genetic abnormalities. Once diagnostic cluster signatures are known, rapid analysis tools for detection of high risk species, strains or mutations can be developed.

Our matching algorithm using signature clusters increases the efficiency of matching HTS reads against data sets of reference genetic material compared to string alignment methods (orders of magnitude faster than BLAST) and even outperforms high performance k-mer string search algorithms (such as USEARCH) for realistic environmental sample studies.

The matching algorithm also maintains good precision and recall compared to less sensitive string search methods and even outperforms USEARCH for reasonably high settings of the “maxaccepts” value on data sets with lower error rates (*ε*≤0.03).

The matching algorithm does not rely on a-priory selection of a parameter limiting the result setlength, such as “max_target_seqs” for BLAST or “maxaccepts” for USEARCH, but self-calibrates to the size of the set of matching clusters of each query sequence.

Using cluster signatures improves recall and accuracy of existing in vitro methods of identification, especially for data sets containing closely related genetic material, without needing to rely on a priori hierarchical phylogenetic grouping.

Cluster signatures and the aodp utility can increase the sensitivity and accuracy of PCR-based and DNA hybridization-based experiments compared to traditional methods based on sequence or phylogenetic clade signatures. Cluster signatures can also be used for targeted enrichment-based HTS, developing accurate, sensitive and efficient diagnostic tools for in vivo or *in silico* detection of high-risk pathogens or mutation of genes linked to genetic disorders or tumors, using genomics, genetics and metagenomics sequencing data.

## Footnotes

1 **Signable sequence**: sequence with at least one sequence signature.

2 **Signable clade**: phylogenetic clade with at least one clade signature.

3 Following phylogenetic tree terminology, we call clades with one sequence or with only identical sequences **leaf clades** and clades with more sequences **internal clades**.

4 Since *λ* is generally much smaller than the size of each sequence, the total number of *λ*-mers can be approximated by the number of nucleotides in the database.

5 This may represent the union of reference and query databases covering a single genome, a set of genomes, or a set of single DNA markers or multiple loci shared by different taxonomic lineages.

6
https://unite.ut.ee/sh_files/UNITE_public_20.11.2016.fasta.zip

7 The bootstrapped test set *4MicotoxinsBootstrap* does not provide true negative examples.
